# Supplementary material for: Evolution of the quality of prenatal care in the primary network of Brazil from 2012 to 2018: What can (and should) improve?
Source: PLoS One. 2022 Jan 18;17(1):e0262217. doi: 10.1371/journal.pone.0262217 (PMC8765636; doi:10.1371/journal.pone.0262217)
Supplement: Attachment — Submitted filename: PONE-D-21-11493.docx [file pone.0262217.s001.docx]

PONE-D-21-11493

The authors present the findings of the Brazilian National Program for the Improvement of Access and Quality in Primary Care (PMAQ) reviews of primary care antenatal services at 3 time points between 2012 and 2018. On-going evaluation of public services to which this analysis contributes is important for improvements in quality of care. However, this manuscript is conceptually and methodologically unclear and not suitable for publication is its present form.

The overall style is not clear and there are some grammatical errors. This is possibly due to translation into English.

Introduction:

I suggest contextualizing the study in Brazil’s antenatal services and health care structures at the time of the reviews. You have not made clear that the PMAQ is a pay for performance scheme and how this works (also noted by reviewer 2); you should distinguish between the different service packages. I would not previous review findings and existing regional differences.

Methods:

As noted by reviewers 2 and 3, many methodological details are absent. The methodology is partially described. It would be useful to have a summary or table of the relevant antenatal care guidelines.

While each review may be cross-sectional, the analysis is more longitudinal at the 3 time points.

Please motivate for the selection of the 6 outcomes and the exclusion of others. At which antenatal visits were the elements of “physical examination”, “education” and “lab tests” required?

117: what does this sentence mean? Is there a reference? “The indicators used in the analysis were validated by the technical areas of the Ministry of Health.”

120: please use an alternative term to ‘skin colour’. Maybe *ethnicity?* And I suggest dropping the word, ‘yellow’. If these terms are necessary, are they part of the official language of the Dept of Health? If so, I suggest stating that ethnicity was characterised using the formal classification of the Department.

Results

Need to include uncertainty ranges for all measures.

Is the Annual change in Table 2 a risk difference? Between Cycles I and III? Is it the β-value of the regression?

Discussion

There a very small changes in proportion, perhaps the p-value is due to the large numbers? Do the you think the changes are clinically or programmatically significant?

What has the addition of Cycle III data added to that already published? The changes between cycles II and III are small and maybe the less interesting observations in the study.

Perhaps you could discuss why some services are well-provided an others not? Per region?

You have not made clear that the PMAQ is a pay for performance scheme. This certainly limits the generalization of the findings to services outside the PMAQ scheme.

Please review and correct the references.
