# Supplementary material for: Evolution of the quality of prenatal care in the primary network of Brazil from 2012 to 2018: What can (and should) improve?
Source: PLoS One. 2022 Jan 18;17(1):e0262217. doi: 10.1371/journal.pone.0262217 (PMC8765636; doi:10.1371/journal.pone.0262217)
Supplement: Attachment — Submitted filename: Response to Reviewers.docx [file pone.0262217.s002.docx]

Response to Reviewers

Dear Dr.Emma Kalk

Acadêmica editor / PLOS ONE

In relation to the manuscript **PONE-D-21-11493**, on behalf of the authors, I thank you for your careful review and also for the collaborators. Then, I send the answers to each questioned point, in order to qualify our manuscript. I remain available.

**Response to the academic editor**

**COMMENT**: The overall style is not clear and there are some grammatical errors. This is possibly due to translation into English.

**ANSWER**: We would like these few problems with the language to be pointed out so that we can fix it.

**COMMENT**

Introduction:

I suggest contextualizing the study in Brazil’s antenatal services and health care structures at the time of the reviews. You have not made clear that the PMAQ is a pay for performance scheme and how this works (also noted by reviewer 2); you should distinguish between the different service packages. I would not previous review findings and existing regional differences.

**ANSWER**: We added content about PMAQ in the Introduction and also in Methods.

**COMMENT**

Methods:

As noted by reviewers 2 and 3, many methodological details are absent. The methodology is partially described. It would be useful to have a summary or table of the relevant antenatal care guidelines.

While each review may be cross-sectional, the analysis is more longitudinal at the 3 time points.

**ANSWER**: We added content in Methods.

**COMMENT**

Please motivate for the selection of the 6 outcomes and the exclusion of others. At which antenatal visits were the elements of “physical examination”, “education” and “lab tests” required?

**ANSWER**: We rewritten the methods; there was no mention in the instruments to which prenatal consultations the elements were questioned; the question was, for example: "In the prenatal consultation(s), did the health professionals examine your mouth?"

**COMMENT**

117: what does this sentence mean? Is there a reference? “The indicators used in the analysis were validated by the technical areas of the Ministry of Health.”

**ANSWER**: The text has been modified to make it clearer.

**COMMENT**

120: please use an alternative term to ‘skin colour’. Maybe *ethnicity?* And I suggest dropping the word, ‘yellow’. If these terms are necessary, are they part of the official language of the Dept of Health? If so, I suggest stating that ethnicity was characterised using the formal classification of the Department.

**ANSWER:** Changes made to the text and table 1

**COMMENT**

Results

Need to include uncertainty ranges for all measures.

Is the Annual change in Table 2 a risk difference? Between Cycles I and III? Is it the β-value of the regression?

**ANSWER:** Confidence intervals are already presented for the main measure (annual change); the measure is not a risk difference, it is an average rate of change between cycles, the β-value of the regression, and represents the increase or decrease in percentage points; for example, for the first indicator, of six or more appointments, the average rate was negative, there was an average decrease of 2.25 percentage points between 2012 and 2018 in the proportion of women who had six or more medical appointments during pregnancy.

**COMMENT**

There a very small changes in proportion, perhaps the p-value is due to the large numbers? Do the you think the changes are clinically or programmatically significant?

**ANSWER:** The changes reflect the actions taken by the health teams, within the scope of their possibilities and incentives received as a result of the Program. It is believed that the PMAQ was able to induce important improvements in almost all investigated indicators, especially in the teams' work process. This can have both clinical and programmatic impact. Small changes in proportions that were already high were expected

**COMMENT**

What has the addition of Cycle III data added to that already published? The changes between cycles II and III are small and maybe the less interesting observations in the study.

**ANSWER:** The inclusion of data from cycle III marks the end of the program and points to the continued growth that would be expected with the continuity of the PMAQ.

**COMMENT**

You have not made clear that the PMAQ is a pay for performance scheme. This certainly limits the generalization of the findings to services outside the PMAQ scheme.

**ANSWER:** The PMAQ was not a typical pay-for-performance program as the financial resources were not transferred directly to professionals, as in other countries, but were transferred to municipal managers.

**Response to the reviewer 2.**

**COMMENT**

Introdução: Incluir as recomendações do PHPN e Rede Cegonha para qualificação do Pré-natal no Brasil. Tornar mais clara a situação do PMAQ (não mais vigente), já que desde dezembro de 2019 foi instituído um novo modelo de financiamento da APS brasileira, o Programa Previne Brasil pela Portaria nº 2.979.

**ANSWER**

As recomendações do PNPH e da Rede Cegonha foram incluídas; também foram esclarecidos aspectos sobre o fim do PMAQ e sua substituição pelo Programa Previne Brasil.

**COMMENT**

Materiais e Métodos: Quais os critérios de inclusão e exclusão adotados? Também senti falta de mais detalhes sobre a amostragem e recrutamento das mulheres: Qual o local das entrevistas e de que forma foram abordadas? Houve estudo piloto do instrumento de coleta? Como as 41 instituições de ensino superior se organizaram para a coleta de dados? Foi realizado cálculo amostral? Em relação aos aspectos éticos, houve utilização do TALE às menores de 18 anos, além do TCLE para seu responsável legal?

**ANSWER:** Todas as respostas a estas perguntas foram acrescidas na seção de Métodos.

**COMMENT**

Discussão: Senti falta de discutir alguns dos resultados do estudo ... e de trazer mais comparações dos achados com outros estudos nacionais e internacionais ... Ademais, como os autores entendem as diferenças das Regiões brasileiras nos resultados do pré-natal?

**ANSWER**: Não foram localizados resultados comparáveis em outros estudos nacionais e internacionais. As diferenças regionais são decorrentes de históricas desigualdades socioeconômicas que afetam o sistema de saúde como um todo. Foram acrescentadas referências para estes resultados.

**COMMENT**

*Em um dos parágrafos da discussão, faz-se necessário melhorar a descrição do novo modelo de financiamento da APS no Brasil. O Programa Previne Brasil realizará repasses financeiros com base em três critérios: a) captação ponderada (cadastros das pessoas), b) pagamento por desempenho (sete indicadores, que são distribuídos em pré-natal, saúde da mulher, saúde da criança e doenças crônicas e avaliados quadrimestralmente) e c) incentivo para ações estratégicas. Em relação ao pagamento por desempenho, o repasse dependerá dos resultados alcançados por cada equipe em um conjunto inicial de sete indicadores, dentre os quais os 3 primeiros são referentes ao pré-natal. Foi mantido o início precoce (até a 20ª semana), realização de 6 ou mais consultas, dos exames para sífilis e HIV e o atendimento odontológico na gestação.

**ANSWER**: Foi melhorada a descrição do Programa Previne.

**COMMENT**

Conclusões: Entendo ser importante acrescentar sugestões de possíveis estratégias que a APS poderia adotar para melhoria da qualidade do pré-natal no Brasil. O que os autores visualizam que ainda poderia ser feito, em termos de gestão interfederativa e de assistência às gestantes brasileiras, para garantia de um pré-natal qualificado?

**ANSWER**: Nossas conclusões já apontam estas sugestões.

**Response to the reviewer 3**

**COMMENT**

The stated purpose of the paper is to “describe the temporal evolution of the coverage and quality of prenatal care performed in primary care in Brazil…” but key concepts like quality are not well articulated or defined. There is no clear discussion in the introduction of what the authors mean by quality (at the moment there is a brief mention of quality on line 61 that is followed directly by a reference to access) and then very little in the methods to show how these concepts were reflected in study design, data collection and analysis. A clearer explanation of the PMAQ and the indicators developed is needed to contextualise the study and provide information for the reader.

**ANSWER**: We've added arguments in the introduction and methods to fulfill this request. References for this conceptual contribution were also added.

**COMMENT**

Regarding the methods, what is the sampling approach and how were participants selected? What proportion of the eligible population does this sample represent in each of the districts and therefore how can we interpret the meaning of these findings?

**ANSWER:** We've added answers to these questions in the Methods section.

**COMMENT**

I am confused by statements like: Line 107 “A total of six dichotomous outcomes (no; yes) were selected” because I am unclear how this statement relates to the previous paragraph which refers to interviews. And again, Line 113 "Four educational guidelines were 113 evaluated (no, yes)".

**ANSWER:** A new wording was made to clarify these doubts.

**COMMENT**

Line 118 "The indicators used in the analysis were validated by the technical areas of the Ministry of Health. – It would be useful to have more details about this.

**ANSWER:** The sentence that generated the doubt was deleted and other explanations were added.

**COMMENT**

Sentence beginning Line 119 does not seem to be finished. Needs restating for clarity.

**ANSWER:** Corrected

**COMMENT**

124 - ‘prevalence of outcomes’ was calculated. What does this mean?

**ANSWER:** A new wording was made to clarify these doubts.

**COMMENT**

Until the conceptual and methodological issues are clarified, I do not think I can provide useful comments on the discussion. At present, parts of the discussion that refer to explanations for change in indicators over time such as the paragraphs beginning on LIne 286 and Line 319 require references.

**ANSWER:** References have been added in both paragraphs.
