# Supplementary material for: Evolution of the quality of prenatal care in the primary network of Brazil from 2012 to 2018: What can (and should) improve?
Source: PLoS One. 2022 Jan 18;17(1):e0262217. doi: 10.1371/journal.pone.0262217 (PMC8765636; doi:10.1371/journal.pone.0262217)
Supplement: Attachment — Submitted filename: Response to Reviewers 2021dec19.docx [file pone.0262217.s003.docx]

Response to Reviewers

Dear Dr.Emma Kalk

Acadêmica editor / PLOS ONE

In relation to the manuscript PONE-D-21-11493, on behalf of the authors, I send the answers to each questioned point in the Minor Queries, in order to qualify our manuscript.

COMMENT: line 140: what is TALE? The abbreviation has not been spelled out or explained.

ANSWER: TALE means free and informed consent term; is the term corresponding to the consent form for minors under 18; the sentence was dropped as it was not really necessary. Information about informed consent is already in the last paragraph of the Methods section: “All participants signed the Informed Consent Form”.

COMMENT: line 159 and Table 1: please delete the descriptor, 'yellow'. Indigenous is sufficiently clear.

ANSWER: The descriptor 'yellow' was deleted in the text and Table 1.

COMMENT: Tables: please indicate that 'Annual % change' is average annual % change.

ANSWER: In the tables 2 and 3 the expression was corrected to “average annual % change”

Best regards

Elaine Tomasi
